# Chloroplast Phylogenomic Analyses Resolve Multiple Origins of the *Kengyilia* Species (Poaceae: Triticeae) *via* Independent Polyploidization Events

**DOI:** 10.3389/fpls.2021.682040

**Published:** 2021-08-06

**Authors:** Shiyong Chen, Hao Yan, Lina Sha, Ning Chen, Haiqin Zhang, Yonghong Zhou, Xing Fan

**Affiliations:** ^1^College of Animal and Veterinary Sciences, Southwest Minzu University, Chengdu, China; ^2^Triticeae Research Institute, Sichuan Agricultural University, Chengdu, China; ^3^Key Laboratory of Crop Genetic Resources and Improvement, Ministry of Education, Sichuan Agricultural University, Ya’an, China

**Keywords:** polyploid, Triticeae, *Kengyilia*, multiple origins, maternal donor

## Abstract

*Kengyilia* is a group of allohexaploid species that arose from two hybridization events followed by genome doubling of three ancestral diploid species with different genomes St, Y, and P in the Triticeae. Estimating the phylogenetic relationship in resolution of the maternal lineages has been difficult, owing to the extremely low rate of sequence divergence. Here, phylogenetic reconstructions based on the plastome sequences were used to explore the role of maternal progenitors in the establishment of *Kengyilia* polyploid species. The plastome sequences of 11 *Kengyilia* species were analyzed together with 12 tetraploid species (PP, StP, and StY) and 33 diploid taxa representing 20 basic genomes in the Triticeae. Phylogenomic analysis and genetic divergence patterns suggested that (1) *Kengyilia* is closely related to *Roegneria*, *Pseudoroegneria*, *Agropyron*, *Lophopyrum*, *Thinopyrum*, and *Dasypyrum*; (2) both the StY genome *Roegneria* tetraploids and the PP genome *Agropyron* tetraploids served as the maternal donors during the speciation of *Kengyilia* species; (3) the different *Kengyilia* species derived their StY genome from different *Roegneria* species. Multiple origins of species *via* independent polyploidization events have occurred in the genus *Kengyilia*, resulting in a maternal haplotype polymorphism. This helps explain the rich diversity and wide adaptation of polyploid species in the genus *Kengyilia*.

## Introduction

Polyploidy, defined as the possession of two or more sets of homologous chromosomes following whole-genome duplication, is a major mechanism in plant evolution and speciation (Otto, 2007; Spoelhof et al., 2017). Recent studies even suggested that multiple origins (including independent origin) of polyploid species are the rule rather than the exception (Soltis and Soltis, 2000; Symonds et al., 2010; Fan et al., 2013; Sha et al., 2017a). Polyploidy promotes variability through the change in the chromosomal number *per se*, increased genetic diversity, and genomic reorganization, leading to benefits in new phenotypes and evolutionary innovation in physiological and ecological flexibility (Ramsey and Ramsey, 2014; Sha et al., 2017b). However, a clear and appropriate identification of phylogenetic relationships among taxa and genomes is needed (Fan et al., 2013).

*Kengyilia* Yen et J. L. Yang, a polyploid perennial genus in the wheat tribe (Poaceae: Triticeae), includes about 22 perennial species that were distributed in a wide range of natural habitats over the upper and middle mountain ranges of Central Asia and the Qinghai-Tibetan Plateau (Yang et al., 1992). *Kengyilia* was also often classified in *Elymus* L. sensu lato, which was the largest taxon in Triticeae, including *Roegneria*, *Kengyilia*, and *Elymus* sensu stricto (Löve, 1984). A comparison of the morphological features among the genera *Kengyilia*, *Roegneria*, *Elymus*, and *Agropyron* suggested that species in *Kengyilia* are intermediate between the species of *Roegneria* C. Koch and *Agropyron* Gaertn., but with some distinct morphological divergence in the spikelet characters between *Kengyilia* and *Roegneria* and between *Kengyilia* and *Agropyron* (Baum et al., 1995). *Kengyilia* species exhibit variation with high (*K. grandiglumis*) to low (*K. thoroldiana*) plants, lax (*K. rigidula*) to dense (*K. hirsuta*) spikes, adnate (*K. longilumis*) to incohesive (*K. stenachyra*) spikelets attached to the rachis, and yellow (*K. gobicola*) to black (*K. melanthera*) anthers.

All the species of *Kengyilia* are allohexaploids (2*n* = 6*x* = 42) with StYP genomes (Yang et al., 1992; Yen et al., 2006). The St and P genomes have originated from *Pseudoroegneria* (Nevski) Á. Löve and *Agropyron* Gaertn., respectively (Löve, 1984). It is unknown where the Y genome originates, although it is a fundamental *Kengyilia* genome (Yen et al., 2006; Fan et al., 2013). Cytogenetic evidence suggested that speciation of the *Kengyilia* polyploid was derived from hybridization between tetraploid *Roegneria* species (2*n* = 4*x* = 28, StY) and diploid *Agropyron* species (2*n* = 2*x* = 14, P) (Yen et al., 2006; Fan et al., 2013). Analysis of nuclear single-copy *Pgk*1 gene sequences suggested that *Kengyilia* species from Central Asia and the Qinghai-Tibetan Plateau have independent origins with geographically differentiated P genome donors (Fan et al., 2012). Data from chloroplast *trn*L-F, *mat*K, *rbc*L, *trn*H-*psb*A, and mitochondrial *Cox*II suggested that different species of *Kengyilia* have derived their maternal lineages either from the species of *Pseudoroegneria* or the species of *Agropyron* or an unknown donor (Zhang et al., 2009; Zeng et al., 2010; Luo et al., 2012). Analysis of *trn*L-F, *mat*K, and *rbc*L sequences showed that four species of *Kengyilia* (*K. kokonorica*, *K. melanthera*, *K. mutica*, and *K. thoroldiana*) were related to species of *Agropyron*, and the remaining sampled species were close to species of *Pseudoroegneria* (Zhang et al., 2009; Luo et al., 2012). In the *trn*H-*psb*A tree, four species of *Kengyilia* (*K. grandiglumis*, *K. hirsuta*, *K. laxiflora*, and *K. rigidula*) were grouped with *Agropyron*, which is inconsistent with the maternal relationship presented by the *trn*L-F, *mat*K, and *rbc*L sequence data (Zhang et al., 2009; Luo et al., 2012). Moreover, analysis of *Cox*II suggested that some species of *Kengyilia* (e.g., *K. batalinii*), *Agropyron*, and *Pseudoroegneria* formed a paraphyletic grade with zero-length branches (Zeng et al., 2010). While these studies added to our understanding of the phylogenetic relationships of *Kengyilia*, the molecular phylogenies based on published chloroplast DNA (*trn*L-F, *mat*K, *rbc*L, and *trn*H-*psb*A) and mitochondrial sequences in resolution of the maternal lineages of *Kengyilia* species are still in dispute either due to the unresolved gene tree with polytomies or incongruence among the cytoplasmic gene data (Zhang et al., 2009; Zeng et al., 2010; Luo et al., 2012). Moreover, the processes that have driven polyploid diversification and speciation, especially with regard to which tetraploid and diploid species, as maternal progenitors, were involved in the hexaploid evolution in *Kengyilia*, remain unclear. Thus, to better understand the maternal contribution to the evolution of the species of *Kengyilia*, it is essential to conduct a good comparative study of chloroplast genome-wide in *Kengyilia* and its relatives, covering nearly all of the genomic combinations in Triticeae.

By integrating 38 newly and 18 previously sequenced plastomes representing the StYP genomes and its related tetraploid and diploid genomic types in Triticeae, this study applies phylogenetic reconstruction methods in combination with an estimate of the genetic distance among the coding region to clarify maternal lineage relationships. Our objectives are to demonstrate a phylogenomic framework for illustrating the maternal donor of *Kengyilia* polyploids and to explore the role of maternal progenitors in the establishment of *Kengyilia* polyploids.

## Materials and Methods

### Plant Materials

A total of 23 polyploids, comprising 11 *Kengyilia* (StYP genomes) species, eight *Agropyron* (PP) tetraploids, one *Douglasdeweya* (StP genomes) species, and three *Roegneria* (StY genomes) species, were analyzed together with 33 diploid taxa representing 20 basic genomes in the Triticeae (Supplementary Table 1). *Brachypodium distachyon* was used as an outgroup. The chloroplast genome sequences of *Triticum–Aegilops* complex, *Secale*, *Pseudoroegneria cognata*, *Pseudoroegneria stipifolia*, *Pseudoroegneria strigosa*, and *Pseudoroegneria tauri* were from the published data (Gornicki et al., 2014; Bernhardt et al., 2017). The remaining sequences in Supplementary Table 1 in Triticeae were newly sequenced. Sample information including accession numbers, origins, genome type, ploidy, and GenBank accession data were also listed in Supplementary Table 1. The seed materials with PI and W6 numbers were graciously provided by the American National Plant Germplasm System (Pullman, WA, United States). The seed materials with ZY and Y numbers were collected by the authors of this article. The plants used for sequencing and voucher specimens are deposited at the Herbarium of Triticeae Research Institute, Sichuan Agricultural University, China (SAUTI).

### Plastome Sequencing and Data Assembly

DNA extractions were performed on young leaves dried in silica using the DNeasy Plant Mini kit (QIAGEN, Valencia, CA, United States) according to the instructions of the manufacturer after homogenization with liquid nitrogen. The plastomes were amplified in overlapping fragments using the long-range PCR method of Yang et al. (2014). The PCR products were fragmented into short inserts (400–600 bp) to construct the sequencing paired-end library according to the NEBNext^®^ protocol. DNA from each individual was indexed using tags and pooled together in one lane of an Illumina Hiseq 4000 PE150 for sequencing. The raw reads were trimmed using Fastp v0.20.0 (Chen et al., 2018) and the clean reads were subjected to *de novo* assembly using NOVOPlasty 4.0 (Dierckxsens et al., 2016) with default parameters. Approximately, 100 million reads were randomly selected and aligned to the close-related sequences of *Agropyron cristatum* using BWA v0.7.15 (Li and Durbin, 2009) (MEM algorithm), and a perfect matched read to the *psb*A gene was selected as the seed input. NOVOPlasty produced two optional sequences which were simply different in small single-copy region (SSC) direction. The sequence with the same SSC direction to *Agropyron cristatum* (GenBank No. KY126307) and *Pseudoroegneria libanotica* (GenBank No. KX822019) was selected and subjected to annotation.

### Sequence Alignment and Analysis

The complete chloroplast sequences were aligned with MAFFT v. 7 (Katoh and Standley, 2013) using the default settings. All alignments were visually inspected in MEGA 6.0 (Tamura et al., 2013) and manually adjusted where needed. We also conducted a co-linear analysis using the software LASTZ, and the results were visualized using AliTV (Ankenbrand et al., 2017). Multiple alignment of the protein-coding sequence was conducted using ClustalW in MEGA 6 (Tamura et al., 2013), with manual adjustment. Amino acid translations were used to guide the nucleotide alignments. The sequence statistics, including nucleotide substitutions, Kimura 2-parameter (K2-p) distances, transition/transversion ratio, and variability of the sequences, were calculated by MEGA 6 (Tamura et al., 2013).

To estimate the genetic differentiation of the protein-coding sequences between *Kengyilia* and its close relatives, the K2-p model was used to calculate the genetic distances of the protein-coding sequences of 52 genes (76 unique genes, excluding 24 which have no variable nucleotide sites and/or are <200 bp). A total of 1,664 (32 samples and 52 protein-coding genes) genetic distances were used to estimate the genome relationship employing hierarchical agglomerative clustering in R (Version 3.4.2; Vienna, Austria). The Hopkins statistic was used for the evaluation of the clustering tendency. The optimal number of clusters was determined by the “fviz_dend” algorithm in the R package “factoextra” Version 1.0.5. Bivariate cluster (*k*-means clustering) analysis based on genetic distances and agglomerative hierarchical clustering was performed by the “clustplot” function in the R package “cluster” package (Version 2.0.6).

### Phylogenetic Analysis

Because complete chloroplast genome sequences offer the greatest phylogenetic resolution (Ma et al., 2014), phylogenomic trees were generated from the complete genomes of all sampled chloroplasts. Phylogenomic analyses were conducted using maximum likelihood (ML) and Bayesian inference (BI). The ML analysis was performed using the IQ-Tree software.^1^ The TVM + F + R3 model was selected for the ML analysis according to ModelFinder implemented in IQ-Tree. To assess branch support, the IQ-Tree analyses used the ultrafast bootstrap approximation (UFboot) with 10,000 replicates (Minh et al., 2013) and the SH-like approximate likelihood ratio test (SH-aLRT) with 1000 bootstrap replicates (Guindon et al., 2010).

The evolutionary model used for BI analysis was determined using ModelTest v3.7 (Ronquist et al., 2012) with Akaike information criterion (AIC). The BI analysis was performed using MrBayes v3.2 (Posada and Crandall, 1998) under the GTR + G + I model that was identified as the best fit by ModelTest. Four Markov Chain Monte Carlo (MCMC) chains (one cold and three heated), applying MrBayes default heating values (*t* = 0.2), were run 1,000,000 generations for plastome data, with each sampled in each data set every 100 generations. Majority-rule (>50%) consensus trees were generated with a relative burn-in of 25%. The statistical confidence in nodes was estimated by posterior probabilities (PP). A PP-value less than 90% was not included in the figures.

## Results

### Characteristics of Chloroplast Genomes and Genes

All sequenced genomes are very similar to the published chloroplast genomes of Triticeae (Gornicki et al., 2014; Bernhardt et al., 2017) and are rather conservative in genome structure and gene content. Their genome size ranges from 134,985 in *Roegneria ciliaris* to 135,489 bp in *A. cristatum* (ZY09064). All plastomes exhibited a typical quadripartite structure that included a pair of IRs separated by a large single-copy region (LSC) and a SSC and contained a total of 109 genes (including 76 protein coding genes, 29 tRNA genes and 4 rRNA genes). Assemblies in the genus *Kengyilia* averaged 135,113 bp, with an estimated 0.064% insertion data (compared to *P*. *libanotica* reference); genus *Roegneria* assemblies averaged just less than 135,079 bp (0.039% estimated insertion data, compared to *P*. *libanotica* reference).

**FIGURE 1:**
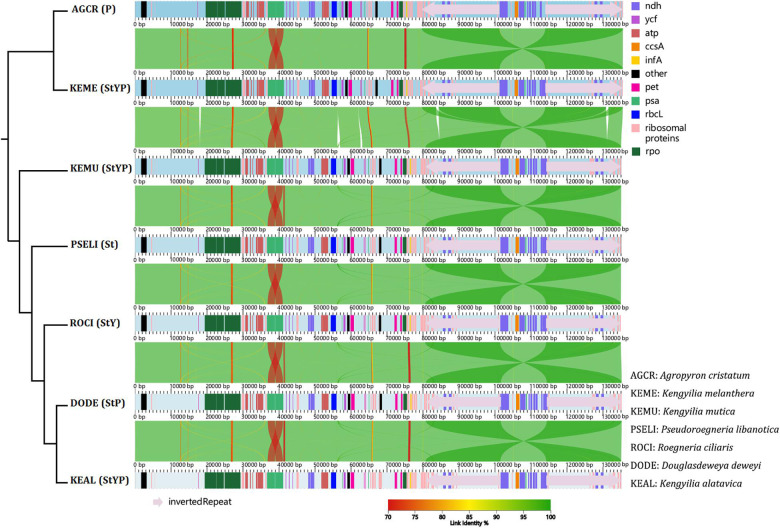
The co-linear analysis of the chloroplast genomes of *Kengyilia* species and its close relatives. The phylogenetic tree was constructed based on the complete chloroplast genomes using ML.

Analysis of co-linearity is inferred for two diploid taxa representing St and P genomes, one tetraploid species with the StP genome, one tetraploid species with the StY genome, and three hexaploid species with the StYP genome (Figure 1). Despite a high degree of co-linearity among these genomes due to the conservation in chloroplast genome structure and gene content, five big indels (at positions 17,819–18,278, 56,172–56,963, 62,664–63,130, 83,590–84,338, and 130,804–131,592 bp, respectively) were detected between the St- and P-containing lineages, which is indicative of high genetic divergence between them.

The features of each of the 76 protein-coding genes in the diploid–polyploid plastome data are summarized in Supplementary Table 2. The lengths of each gene ranged from 90 (*pet*N) to 4,440 (*rpo*C2) bp. The proportion of the variable sites (variable sites/total sites, V/T) varied from 0 (e.g., *pet*G) to 3.36% (*rpl*32). The ratio of parsimony-informative characters per total aligned characters was greatest for *pet*L (2.08%) and lowest for *pet*G, *psb*F, and *rpl*23 (0).

### Phylogenetic Analyses

Bayesian phylogenetic reconstruction of the plastome data under the GTR + G + I model resulted in a tree with high posterior probability support across most clades. The ML analyses in IQ-Tree under the TVM + F + R3 model recovered the same topology as the Bayesian analyses (Supplementary Figure 1).

**FIGURE 2:**
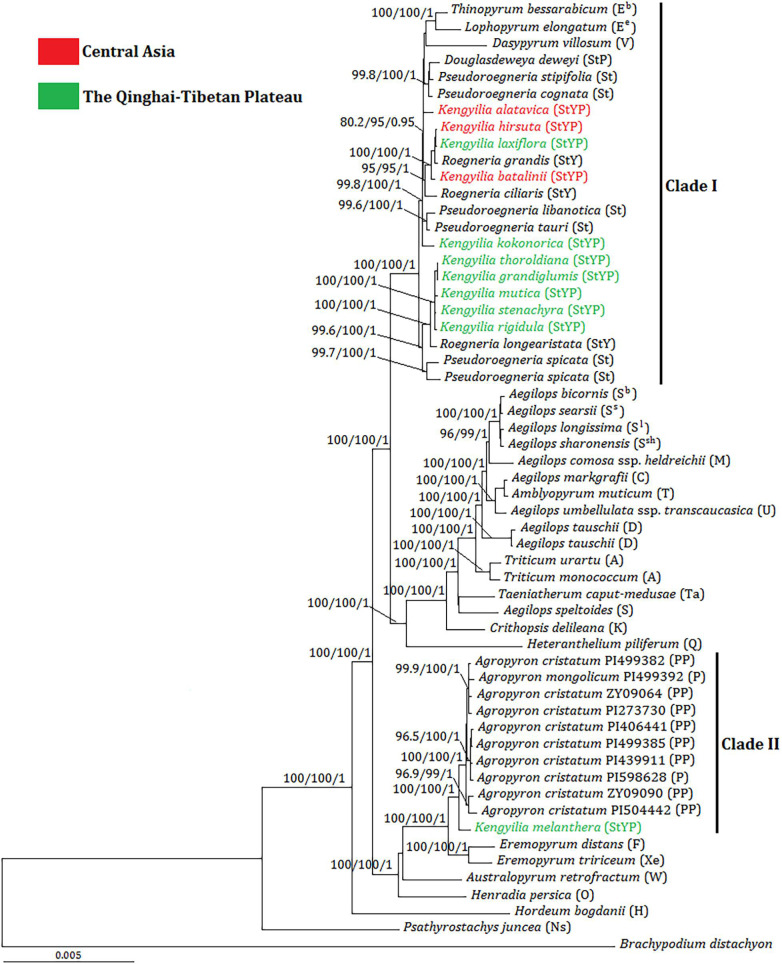
Bayesian tree inferred from the complete chloroplast genome sequences for *Kengyilia* species and its diploid relatives in the Triticeae. Numbers above branches are values of the statistical support values, and values are indicated only if deemed robust as follows: UFboot ≥ 95%/SH-aLRT ≥ 80%/PP ≥ 0.9. The capital letters in brackets indicate the genome type of the species. Different colors indicated the geographic information of *Kengyilia* species.

The tree illustrated in Figure 2 was the BI tree with statistical support (UFboot, SH-aLRT, and PP) above branches. The phylogenetic tree showed that plastome sequences of *Kengyilia* were split into two major clades (Clade I and II) with consistent statistical support (100% UFboot and SH-aLRT; 1.0 PP). The Clade I included *Thinopyrum* (E^b^), *Lophopyrum* (E^e^), *Dasypyrum* (V), and *Pseudoroegneria* (St), as well as all the sampled St-containing (*Douglasdeweya*, StP; *Roegneria*, StY; and *Kengyilia*, StYP) polyploid species (except for *K. melanthera*), which also had the consistent statistical support (100% UFboot and SH-aLRT; 1.0 PP). In this clade, *Thinopyrum*, *Lophopyrum*, *Dasypyrum*, *Douglasdeweya*, four species of *Pseudoroegneria* (*P. stipifolia*, *P. cognata*, *P. Libanotica*, and *P. tauri*), two species of *Roegneria* (*R. grandis* and *R. ciliaris*), and four species of *Kengyilia* (*K. alatavica*, *K. hirsuta*, *K. laxiflora*, and *K. batalinii*) were in one subclade (99.8% UFboot, 100% SH-aLRT, and 1.0 PP). *K. alatavica* from Central Asia formed a paraphyletic grade with *Thinopyrum*, *Lophopyrum*, *Dasypyrum*, *Douglasdeweya*, and two species of *Pseudoroegneria* (*P. stipifolia* and *P. cognata*). Two *Kengyilia* species from Central Asia (*K. hirsuta* and *K. batalinii*) and one *Kengyilia* species from the Qinghai-Tibetan Plateau (*K. laxiflora*) were clustered with two species of *Roegneria* (*R. grandis* and *R. ciliaris*) (95% UFboot, 95% SH-aLRT, and 1.0 PP). *K. kokonorica* from the Qinghai-Tibetan Plateau and two species of *Pseudoroegneria* (*P. libanotica* and *P. tauri*) formed a paraphyletic grade in the subclade. Five species of *Kengyilia* from the Qinghai-Tibetan Plateau (*K. thoroldiana*, *K. grandiglumis*, *K. mutica*, *K. stenachyra*, and *K. rigidula*) were grouped with one species of *Roegneria* (*R. longearistata*) (100% UFboot, 100% SH-aLRT, and 1.0 PP), and this group was a sister group to two accessions of *Pseudoroegneria spicata* (99.6% UFboot, 100% SH-aLRT, and 1.0 PP). The clade II contained all sampled *Agropyron* species (*A. cristatum* and *A. mongolicum*) and *K. melanthera* from the Qinghai-Tibetan Plateau (100% UFboot, 100% SH-aLRT, and 1.0 PP).

### Statistic of K2-p Distance Matrix

**FIGURE 3:**
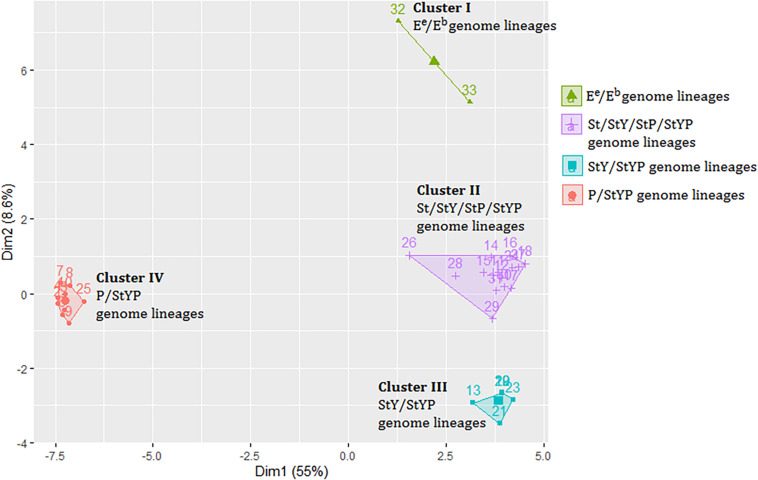
The cluster plot based on a distance matrix of *Kengyilia* species and its close relatives. Numbers represent lineages listed in Supplementary Table 3.

A distance matrix including 1,664 genetic values was generated to investigate the relationship between the plastomes of *Kengyilia* and those of its close relatives (Supplementary Table 3). The Hopkins statistic was found to be 0.2057, indicating that data provides information to cluster the samples. Analysis of hierarchical agglomerative clustering shows four major clusters (Figure 3), which correspond to the four genomic types (P/StYP, E^e^/E^b^, StY/StYP, and St/StP/StY/StYP). This is also well congruent with the groupings in the phylogenomic tree inferred from the plastome data including all sampled Triticeae plants.

## Discussion

The cpDNA-based (*trn*L-F, *mat*K, *rbc*L, and *trn*H-*psb*A) phylogenies of the genus *Kengyilia*, especially with regard to the origin of the maternal donor during hexaploid polyploidization events, were largely unresolved due to the occurrence of many polytomies and incongruence among the published gene trees (Zhang et al., 2009; Luo et al., 2012). Ma et al. (2014) pointed out that despite missing samples, phylogenetic analysis of plastome sequences can offer the greatest phylogenetic resolution. In this study, a resolved tree with high statistical support was inferred from the plastome sequences of *Kengyilia* and those of its relatives in Triticeae, allowing the relationship regarding the maternal lineages of *Kengyilia* to be clarified.

In the phylogenomic tree, 10 species of *Kengyilia* (*K. alatavica*, *K. hirsuta*, *K. laxiflora*, *K. batalinii*, *K. kokonorica*, *K. thoroldiana*, *K. grandiglumis*, *K. mutica*, *K. stenachyra*, and *K. rigidula*), *Roegneria*, and *Pseudoroegneria* were in one group with consistent support, indicating that *Pseudoroegneria* is likely to be the maternal donor of these 10 StYP genome *Kengyilia* species and the sampled StY genome *Roegneria* species. Since *Kengyilia* species arose from two hybridization events followed by three genome doublings (the St, Y, and P genomes), with one first generating the StY genome *Roegneria* and the other forming the StYP genome (Yen et al., 2006; Fan et al., 2012); *Roegneria* served as the maternal donor during the speciation of the 10 *Kengyilia* species.

Analysis of *trn*L-F suggested that four species of *Kengyilia* (*K. kokonorica*, *K. melanthera*, *K. mutica*, and *K. thoroldiana*) were closely related to species of *Agropyron* (Zhang et al., 2009). A similar deep-level relationship regarding the maternal lineages is also presented by Luo et al. (2012), although molecular characters (including *mat*K, *rbc*L, and *trn*H-*psb*A) and more taxa were sampled from *Kengyilia*. In this study, only *K. melanthera* was grouped with the species of *Agropyron*, and the remaining three species (*K. kokonorica*, *K. mutica*, and *K. thoroldiana*) were placed into the clade including St-containing species. Moreover, the plastome sequence of *K. melanthera* and *Agropyron* are obviously distinct from those of the St-containing species. Thus, the molecular phylogenies based on the published cpDNA fragments and the present plastome sequence data in resolution of the placement of *K. kokonorica*, *K. mutica*, and *K. thoroldiana* led to contradictory results, apparently. Discordances among the phylogenetic trees result from methodological artifacts (e.g., sampling error and/or a failure of molecular characters) and the complex dynamics of the evolutionary processes in organisms (e.g., hybridization and/or ancestral polymorphisms) (Betancur-R et al., 2014; Sha et al., 2017a). Sampling error is likely to be the candidate for the current incongruences because our samples for the comparative phylogenies with *Kengyilia* species included nearly all of the monogenomic genera accepted in genome-based classifications of the Triticeae, and most monogenomic genera were not covered in the previous study (Zhang et al., 2009; Luo et al., 2012). It is well known that molecular characters can affect the accuracy of phylogenetic estimates (Ma et al., 2014). Incongruences would also be the result of a lack of molecular characters. Fewer molecular characters in cpDNA regions, as indicated by our estimate for the variable features of each chloroplast protein-coding gene (Supplementary Table 3), together with their slowly evolving rates in the chloroplast genome, would not only provide lower variable information for the accuracy of the phylogenetic reconstruction but also result in the occurrence of polytomies in the phylogenetic tree. On the contrary, the plastome data offer enough molecular characters for the accuracy of phylogenetic estimates with a well-supported topology. Both hybridization and ancestral polymorphisms acting alone or in concert can generate discordance and therefore are the principal processes to explain the phylogenetic incongruence in Triticeae species (Mason-Gamer, 2013; Sha et al., 2017a).

**FIGURE 4:**
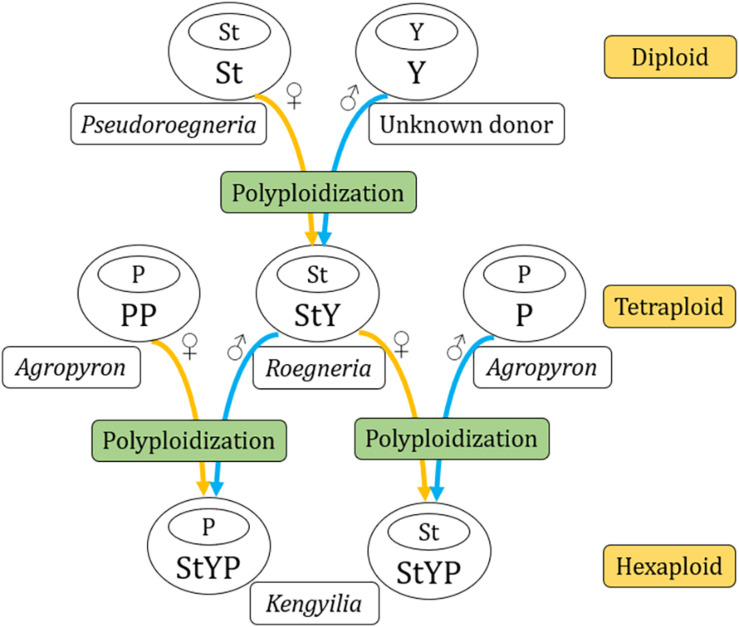
A diagram of lineage relationships among genera *Kengyilia*, *Roegneria*, and *Agropyron*.

The analysis of the genetic distance matrix based on the 52 protein-coding genes suggested that *Lophopyrum* and *Thinopyrum* are closely related to the St-containing species. In the phylogenomic tree inferred from the complete chloroplast genome, *Lophopyrum*, *Thinopyrum*, *Dasypyrum*, and two species of *Pseudoroegneria* (*P. stipifolia* and *P. congnata*) form a monophyletic group. These results indicated *Lophopyrum*, *Thinopyrum*, *Dasypyrum*, and *Pseudoroegneria* (most likely *P. stipifolia* and *P. congnata*) shared ancestral polymorphisms due to the incomplete diversification of the common maternal ancestry. Such ancestral polymorphisms could be genetically transmitted to certain polyploid species (e.g., StP, StY, and StYP) *via* the hybridization between *Pseudoroegneria* as the female parent and the donors with Y and/or P genomes. The hypothesis of hybridization is also a likely candidate to explain the conflict because different polyploid species with the same genotypes could be derived from different parental donors *via* independent hybridization events, generating a diverse array of polyploid genotypes in Triticeae (Sun et al., 2008; Fan et al., 2013). The present plastome data also provides support for the independent origin of certain polyploid species, which can be shown by different *Kengyilia* species that were grouped with different *Roegneria* species in a phylogenetic tree. For example, in the clade I of the phylogenomic tree, three *Kengyilia* species (*K. hirsuta*, *K. laxiflora*, and *K. batalinii*) were clustered with *R. grandis* with strong statistical support (100% UFboot, 100% SH-aLRT, and 1.0 PP), and five *Kengyilia* species (*K. thoroldiana*, *K. grandiglumis*, *K. mutica*, *K. stenachyra*, and *K. rigidula*) were grouped with *R. longearistata* (100% UFboot, 100% SH-aLRT, and 1.0 PP). The analysis of genetic distances based on the 52 protein-coding sequences also presented similar results. Sympatric distribution among *R. grandis*, *R. longearistata*, and *Agropyron* species have provided an opportunity in physical proximity for hybridization events. It is thus suggested that different *Kengyilia* species derived their StY genome from different *Roegneria* species. Our data also indicated that *Agropyron* species served as the maternal donor during the speciation of *K. melanthera*, providing additional support for the independent origins of different *Kengyilia* species. However, it seems unlikely that maternal *Agropyron* lineage in *K. melanthera* resulted from hybridization between high ploidy *Roegneria* species with StY genomes (served as paternal donor) and diploid P genome *Agropyron* species. One possible explanation is that the P genome of *K. melanthera* originated from the tetraploid *Agropyron* lineage as the female parent (Figure 4). Given the present data, multiple origins of polyploid species result in a maternal haplotype polymorphism and could explain the rich diversity and wide adaptation of polyploid species in the genus *Kengyilia* (Yen et al., 2006).

## Conclusion

The present analysis of phylogenetic relationships in *Kengyilia* based on the plastome sequences revealed that both *Roegneria* and *Agropyron* tetraploid species served as the maternal donor during the speciation of *Kengyilia* species, and different *Roegneria* species contributed their StY genome to different *Kengyilia* species. This is indicative of the independent origin of different *Kengyilia* species, which shed new light on our understanding of the maternal lineages, polyploidization events, and speciation process of *Kengyilia*.

## Data Availability Statement

The datasets presented in this study can be found in online repositories. The names of the repository/repositories and accession number(s) can be found in the article/Supplementary Material.

## Author Contributions

XF, SC, and YZ designed the research. SC, HY, LS, and NC performed the research. LS, HZ, and YZ collected and identified the plant materials. XF, SC, and HY analyzed the data and wrote the manuscript. XF edited the manuscript. All authors contributed to the article and approved the final manuscript.

## Conflict of Interest

The authors declare that the research was conducted in the absence of any commercial or financial relationships that could be construed as a potential conflict of interest.

## Publisher’s Note

All claims expressed in this article are solely those of the authors and do not necessarily represent those of their affiliated organizations, or those of the publisher, the editors and the reviewers. Any product that may be evaluated in this article, or claim that may be made by its manufacturer, is not guaranteed or endorsed by the publisher.

## Footnotes

1
http://www.cibiv.at/software/iqtree/
