# Supplementary figures and images for: Chloroplast Phylogenomic Analyses Resolve Multiple Origins of the Kengyilia Species (Poaceae: Triticeae) via Independent Polyploidization Events
Source: Front Plant Sci. 2021 Aug 6;12:682040. doi: 10.3389/fpls.2021.682040 (PMC8377392; doi:10.3389/fpls.2021.682040)

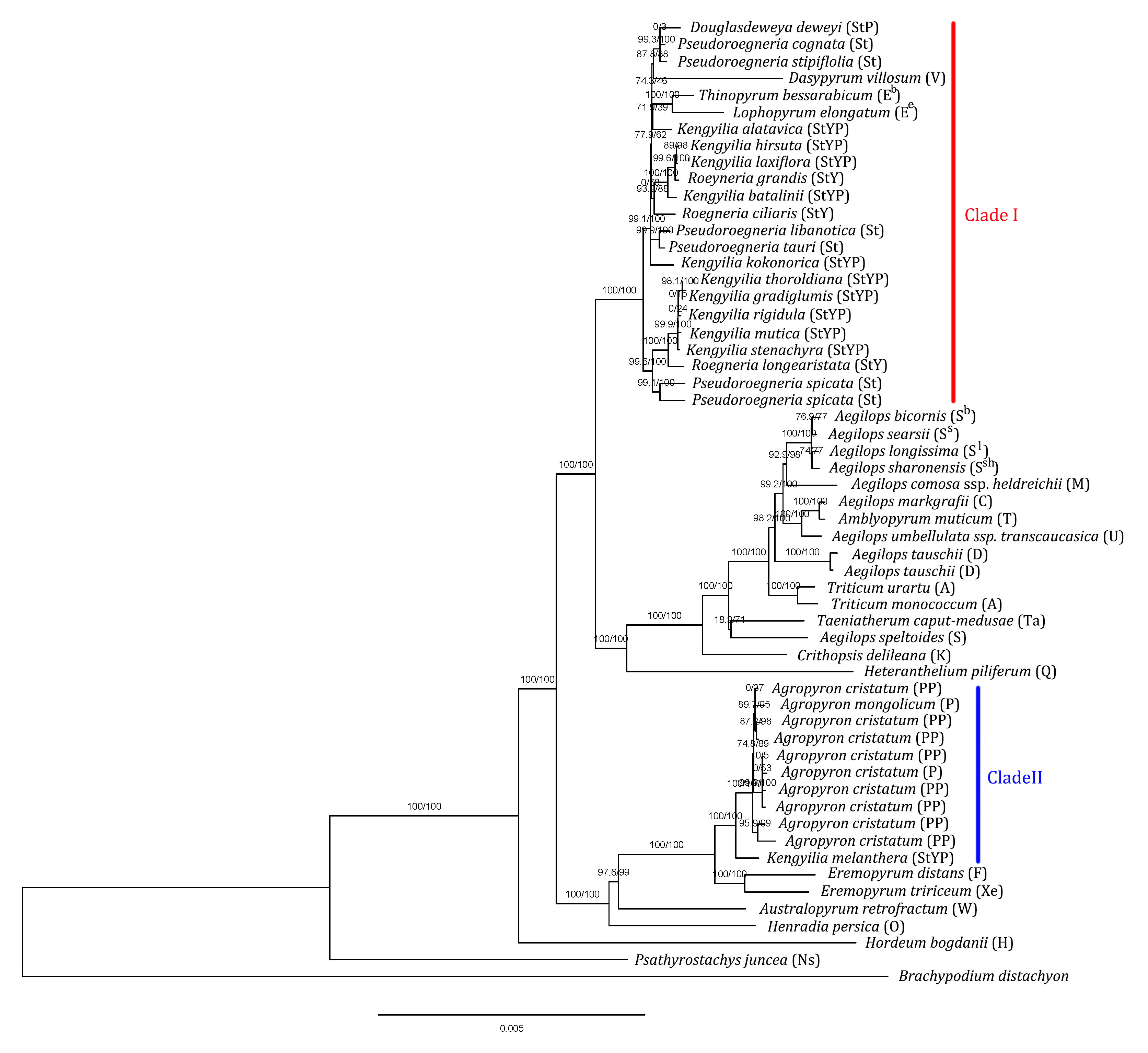

Supplement: Supplementary file 1 [file Image_1.JPEG]
