# Supplementary material for: Chloroplast Phylogenomic Analyses Resolve Multiple Origins of the Kengyilia Species (Poaceae: Triticeae) via Independent Polyploidization Events
Source: Front Plant Sci. 2021 Aug 6;12:682040. doi: 10.3389/fpls.2021.682040 (PMC8377392; doi:10.3389/fpls.2021.682040)
Supplement: Supplementary file 2 [file Table_1.DOC]

Table S1 List of taxa used in this study

| **Species** | **Accession no.** | **Genebank** | **Origin** | **Genome** | **Ploidy** | **Chloroplast genome size** |
| --- | --- | --- | --- | --- | --- | --- |
| ***Aegilops* L.** |  |  |  |  |  |  |
| *Aegilops bicornis* | Cultivar Clae57 | **NC024831** | ND | S^b^ | 2x | 136,861 |
| *Aegilops longissima* | Cultivar TA1924 | **NC024830** | ND | S^l^ | 2x | 136,875 |
| *Aegilops searsii* | Cultivar TA1837 | **NC024815** | Jordan | S^s^ | 2x | 136,863 |
| *Aegilops sharonensis* | Cultivar TA1995 | **NC024816** | Turkey | S^sh^ | 2x | 136,867 |
| *Aegilops speltoides* | SPE0661 | **NC022135** | ND | S | 2x | 113,536 |
| *Aegilops tauschii* | AE429 | **NC022133** | United States of America | D | 2x | 114,112 |
| *Aegilops tauschii* | Cultivar AL8/78 | **KJ614412** | ND | D | 2x | 135,568 |
| *Aegilops comosa* ssp. *heldreichii* | AE 783 | **KY636010** | Balkans | M | 2x | 114,379 |
| *Aegilops markgrafii* | AE 1381 | **KY636033** | NE Mediterranean | C | 2x | 114,884 |
| *Aegilops umbellulata* ssp. *transcaucasica* | AE 1070 | **KY636056** | SE Europe, SW Asia | U | 2x | 114,852 |
| ***Agropyron* J. Gaertn.** |  |  |  |  |  |  |
| *Agropyron cristatum* | PI 598628 | KY126307 | Kazakhstan | P | 2x | 135,554 |
| *Agropyron cristatum* | PI 499382 | MN703653 | Xilinhot, Inner Mongol, China | PP | 4x | 135,486 |
| *Agropyron cristatum* | PI 499385 | MN703657 | Xilinhot, Inner Mongol, China | PP | 4x | 135,454 |
| *Agropyron cristatum* | PI 273730 | MN703654 | Buryatia Russian Federation | PP | 4x | 135,498 |
| *Agropyron cristatum* | PI 439911 | MN703655 | Stavropol Russian Federation | PP | 4x | 135,452 |
| *Agropyron cristatum* | PI 406441 | MN703656 | Former Soviet Union | PP | 4x | 135,453 |
| *Agropyron cristatum* | PI 504442 | MN703668 | Xining, Qinghai China | PP | 4x | 135,465 |
| *Agropyron cristatum* | ZY 09090 | MN703672 | Qinghai, China | PP | 4x | 135,478 |
| *Agropyron cristatum* | ZY 09064 | MN703671 | Qinghai, China | PP | 4x | 135,489 |
| *Agropyron mongolicum* | PI 499392 | MH285848 | Inner Mongal, China | P | 2x | 135,547 |
| ***Amblyopyrum* (Jaub. & Spach) Eig** |  |  |  |  |  |  |
| *Amblyopyrum muticum* | PI 560125 | **KY636075** | Turkey | T | 2x | 114,608 |
| ***Australopyrum* (Tzvelev) Á. Löve** |  |  |  |  |  |  |
| *Australopyrum retrofractum* | PI 533013 | MH331642 | NEW south Wales, Australia | W | 2x | 135,417 |
| ***Crithopsis* Jaub. & Spach** |  |  |  |  |  |  |
| *Crithopsis delileana* | ND | MH285849 | Greece | K | 2x | 136,436 |
| ***Dasypyrum* (Cosson & Durieu) T. Durand** |  |  |  |  |  |  |
| *Dasypyrum villosum* | W6 7264 | MH285850 | Greece | V | 2x | 135,249 |
| ***Douglasdeweya* C. Yen, J. L Yang & B. R. Baum** | |  |  |  |  |  |
| *Douglasdeweya deweyi* | PI 502264 | MK775249 | Russia | StP | 4x | 135,192 |
| ***Eremopyrum* (Ledeb.) Jaub. & Spach** |  |  |  |  |  |  |
| *Eremopyrum tririceum* | Y 206 | MH285852 | Afghanistun | Xe | 2x | 135,554 |
| *Eremopyrum distans* | TA 2229 | MH285852 | Xinjiang, China | F | 2x | 135,589 |
| ***Henrardia* C. E. Hubb.** |  |  |  |  |  |  |
| *Henradia persica* | ND | MH285853 | Iran | O | 2x | 135,659 |
| ***Heteranthelium* Hochst.** |  |  |  |  |  |  |
| *Heteranthelium piliferum* | PI 401352 | MH285854 | Iran | Q | 2x | 136,768 |
| ***Hordeum* L.** |  |  |  |  |  |  |
| *Hordeum bogdanii* | PI 531761 | MH331641 | Xinjiang, China | H | 2x | 136,968 |
| ***Kengyilia* Yen et J. L Yang** |  |  |  |  |  |  |
| *Kengyilia alatavica* | PI 499588 | MN703658 | Kazakhstan | StYP | 6x | 135,060 |
| *Kengyilia hirsuta* | W6 22131 | MN703659 | Xinjiang, China | StYP | 6x | 135,139 |
| *Kengyilia kokonorica* | Y 2880 | MN703660 | Qinghai, China | StYP | 6x | 135,065 |
| *Kengyilia mutica* | PI 531653 | MN703661 | Qinghai, China | StYP | 6x | 135,055 |
| *Kengyilia thoroldiana* | ZY 17047 | MN703662 | Qinghai, China | StYP | 6x | 135,027 |
| *Kengyilia stenachyra* | Y 2723 | MN703663 | Gansu, China | StYP | 6x | 135,049 |
| *Kengyilia gradiglumis* | ZY 17041 | MN703664 | Qinghai, China | StYP | 6x | 135,038 |
| *Kengyilia rigidula* | ZY 17046 | MN703665 | Gansu, China | StYP | 6x | 135,072 |
| *Kengyilia laxiflora* | ZY 17040 | MN703666 | Qinghai, China | StYP | 6x | 135,127 |
| *Kengyilia batalinii* | PI 547361 | MK775255 | Kyrgyzstan | StYP | 6x | 135,107 |
| *Kengyilia melanthera* | ZY 3042 | MN703667 | Sichuan, China | StYP | 6x | 135,500 |
| ***Lophopyrum* (Host) Á. Löve** |  |  |  |  |  |  |
| *Lophopyrum elongatum* | PI 531718 | MH331643 | St. Angulf, France | E^e^ | 2x | 135,020 |
| ***Psathyrostachys* Nevski** |  |  |  |  |  |  |
| *Psathyrostachys juncea* | PI 430871 | MH331640 | Former Soviet Union | Ns | 2x | 136,597 |
| ***Pseudoroegneria* (Nevski) Á. Löve** |  |  |  |  |  |  |
| *Pseudoroegneria cognata* | GRA 1308 | **KY636117** | SW Asia, West Himalaya | St | 2x | 113,480 |
| *Pseudoroegneria spicata* | PI 632532 | **KY636118** | Utah United States | St | 2x | 114,165 |
| *Pseudoroegneria spicata* | PI 232134 | MH285855 | Wyoming United States | St | 2x | 135,165 |
| *Pseudoroegneria stipiflolia* | PI 325181 | **KY636121** | Stavropol Russian Federation | St | 2x | 113,450 |
| *Pseudoroegneria tauri* | PI 401333 | **KY636131** | Iran | St | 2x | 113,496 |
| *Pseudoroegneria libanotica* | PI 228392 | KX822019 | Iran | St | 2x | 135,026 |
| ***Roegneria* C. Koch** |  |  |  |  |  |  |
| *Roegneria grandis* | ZY 3189 | MN703669 | Shanxi, China | StY | 4x | 135,079 |
| *Roegneria longearistata* | Y 0698 | MN703670 | China | StY | 4x | 135,058 |
| *Roegneria ciliaris* | ZY 11004 | MK775252 | Sichuan, China | StY | 4x | 134,985 |
| ***Taeniatherum* Nevski** |  |  |  |  |  |  |
| *Taeniatherum caput-medusae* | PI 220591 | MH285856 | Afghanistan | Ta | 2x | 136,861 |
| ***Thinopyrum* Á. Löve** |  |  |  |  |  |  |
| *Thinopyrum bessarabicum* | PI 531712 | MH331639 | Estonia | E^b^ | 2x | 135,003 |
| ***Triticum* L.** |  |  |  |  |  |  |
| *Triticum monococcum* L. | ND | **LC005977** | ND | A | 2x | 136,886 |
| *Triticum urartu* | PI 428335 | **KJ614411** | Béqaa Lebanon | A | 2x | 136,865 |
| ***Brachypodium* Beauv.** |  |  |  |  |  |  |
| *Brachypodium distachyon* | cultivar Bd21 | **EU325680** | ND | ND |  | 135,199 |

The GenBank accession number with bold represent previously published sequences from the GenBank. ND: not determined. The genome designations are according to Wang et al. (1994). The genome constitution in each species refers to Dewey (1982) and Yen et al (2015).
